# Functionalized chitosan electrospun nanofiber for effective removal of trace arsenate from water

**DOI:** 10.1038/srep32480

**Published:** 2016-08-30

**Authors:** Ling-Li Min, Lu-Bin Zhong, Yu-Ming Zheng, Qing Liu, Zhi-Huan Yuan, Li-Ming Yang

**Affiliations:** 1 CAS Key Laboratory of Urban Pollutant Conversion, Institute of Urban Environment, Chinese Academy of Sciences, Xiamen 361021, China; 2 College of Resources and Environment, University of Chinese Academy of Sciences, Beijing 100049, China; 3 Department of Chemical & Biomolecular Engineering, National University of Singapore, 21 Lower Kent Ridge Road, 119077, Singapore

## Abstract

An environment-friendly iron functionalized chitosan elctrospun nanofiber (ICS-ENF) was synthesized for trace arsenate removal from water. The ICS-ENF was fabricated by electrospinning a mixture of chitosan, PEO and Fe^3+^ followed by crosslinking with ammonia vapor. The physicochemical properties of ICS-ENF were characterized by FESEM, TEM-EDX and XRD. The ICS-ENF was found to be highly effective for As(V) adsorption at neutral pH. The As(V) adsorption occurred rapidly and achieved equilibrium within 100 min, which was well fitted by pseudo-second-order kinetics model. The As(V) adsorption decreased with increased ionic strength, suggesting an outer-sphere complexation of As(V) on ICS-ENF. Freundlich model well described the adsorption isotherm, and the maximum adsorption capacity was up to 11.2 mg/g at pH 7.2. Coexisting anions of chloride and sulfate showed negligible influence on As(V) removal, but phosphate and silicate significantly reduced As(V) adsorption by competing for adsorption sites. FTIR and XPS analysis demonstrated –NH, –OH and C–O were responsible for As(V) uptake. ICS-ENF was easily regenerated using 0.003 M NaOH, and the removal rate remained above 98% after ten successively adsorption-desorption recycles. This study extends the potential applicability of electrospun nanofibers for water purification and provides a promising approach for As(V) removal from water.

Arsenic, existing as various complex forms in the aquatic environment^1^,^2^, has been identified as one of the most harmful and toxic contaminants found in the environment^3^,^4^,^5^,^6^,^7^, because the exposure to arsenic species can lead to lung and bladder cancer, skin lesions, and even death in humans^8^. The sources of arsenic contamination in groundwater are both natural and anthropogenic^2^,^8^. The elevation of arsenic concentration in groundwater has been reported in many countries throughout the world, which has direct consequences to pose great threat to human health. Arsenic poisoning of groundwater was first reported in Taiwan in 1968^2^. It is estimated that there were 19.6 million people at risk of being affected by the consumption of arsenic contaminated groundwater in China^9^. To minimize the health problems associated with arsenic in water, the World Health Organization (WHO) had recommended a strict permissible limit of 10 μg/L as maximum arsenic contaminant level in drinking water^1^.

Various technologies have been developed for arsenic removal, including precipitation, membrane processes, ion exchange, and adsorption^2^. Adsorption has been regarded as the most promising method owing to its cost effectiveness and simplicity in operation^10^,^11^, especially when the arsenic concentration is low. Recently, biosorption has been attracted more attention because the biosorbents are eco-friendly and capable of extracting trace toxic elements from a large volume of solution^1^,^12^,^13^.

Many biosorbents have been reported for arsenic adsorption, such as bacteria cells^14^, plant biomass^15^ and chitosan based materials^16^,^17^. Recently, the development of chitosan based adsorbents for metal ions removal has been becoming a hot topic due to its special physical and chemical properties. Chitosan is a transformed polysaccharide obtained by the deacetylation of chitin and can be easily prepared in many different physical forms such as nanoparticles, gel beads, membranes and fibers^18^,^19^. Chitosan flakes^20^, molybdate-impregnated chitosan beads^21^, chitosan-coated biosorbent^8^, iron coated chitosan flakes^22^, chitosan-immobilized sodium silicate^23^, TiO_2_-impregnated chitosan beads^24^ and magnetic nanoparticles impregnated chitosan beads^25^ have been reported for the adsorptive removal of arsenic from water.

It is well known that larger specific surface area for a particular adsorbent means higher adsorption capacity, and the fabrication of chitosan porous or fibrous structure will definitely enlarge its specific surface area. This could be obtained via electrospinning, an emerging technique to prepare continuous and ultrafine nanofibers^26^,^27^. Nanofibers produced by electrospinning combines the advantages of nanomaterial (high specific surface area) and bulk material (easy separation from water), which has been recently employed as one of the most effective absorbents^28^,^29^.

However, chitosan has rigid D-glucosamine repeat units with regularly arranged hydroxyl and amino groups, which leads to poor electrospinnability^30^. Our previous study indicated that the addition of a compatible polymer, poly(ethylene oxide) (PEO), could enhance the spinnability of chitosan and yield faultless nanofibers^31^. The fabricated chitosan based electrospun nanofiber (CS-ENF) has been employed for arsenic removal. The results indicated that it could be an effective arsenic absorbent at acidic condition, however, the CS-ENF showed little affinity towards As(V) with the circumstance of pH around 7.0^31^. It has been reported that some metal-based material have been incorporated into electrospun nanofibers, which could significantly enhance the composite fibers to achieve desired functional properties^28^. Additionally, iron-based materials were frequently considered as adsorbent for arsenic removal from water under neutral pH^32^,^33^,^34^. Therefore, in this work, Fe^3+^ was selected as the additive to modify the properties of CS-ENF, and the iron functionalized chitosan based elctrospun nanofiber (ICS-ENF) was successfully fabricated in an environment-friendly way. The physicochemical properties and sorption mechanism were systematically explored using a variety of characterization tools, including FESEM, TEM-EDX, XRD, FTIR and XPS. Additionally, the arsenic removal performance of ICS-ENF was examined by both adsorption kinetics and isotherm study, and the influences of various experimental parameters, such as pH, ionic strength and competitive anions were also investigated. Finally, the regeneration and column tests were carried out to verify the potential reusability and reliability.

## Results and Discussion

### Characterization of nanofibers

**Figure 1:**
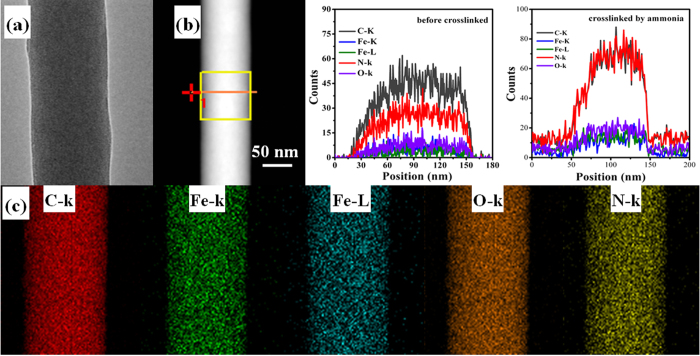
(**a**) TEM image, (**b**) line scan, and (**c**) EDX elemental mappings of C, Fe, O, and N on the surface of ICS-ENF.

The color of chitosan electrospun nanofiber changed from white to light yellow after the addition of FeCl_3_. The morphologies and fiber diameter distributions of both ICS-ENF and CS-ENF are shown in Fig. S1. The FESEM images show that the electrospun nanofibers were beadless, continuous and highly porous, which is also supported by TEM observed in Fig. 1. The fiber diameter distributions demonstrated that the average fiber diameter increased from 128 nm to 153 nm due to the incorporation of iron to chitosan matrix (Fig. S1a,b). The tensile modulus of the chitosan electrospun nanofiber didn’t change with the addition of 3 wt.% FeCl_3_ (Fig. S1c). However, a small decrease in the elongation was observed with the addition of FeCl_3_. Additionally, Fig. 1 represents corresponding elemental analysis of the nanofibers, which indicates that the relative content of nitrogen in ICS-ENF significantly increased after crosslinking by ammonia vapor (Fig. 1b). Furthermore, the EDX elemental mapping (Fig. 1c) demonstrated the fairly uniform distribution of Fe in the entire nanofibers which indicates that iron was successfully impregnated into the chitosan nanofiber.

Figure S2 illustrates the typical XRD patterns of CS-ENF and ICS-ENF. Two characteristic peaks of 2θ between 5 and 25° for CS-ENF were observed. The peak around 2θ = 7.5° was related to chitosan amorphous structure due to the presence of random amino groups (–NH_2_), while the peak in the 2θ region between 17 and 25° was corresponding to the biopolymer structure resulting from packing of the polymer chain and their inter-chain interactions^35^. Similar with CS-ENF nanofiber, ICS-ENF also contains two peaks, but it presented a higher crystallinity than that of CS-ENF in terms of the relatively sharp peak around 2θ = 10.1° (Fig. S2). Moreover, after doped with iron, the peak corresponding to 2θ = 21° was shifted from 20.5° to 22.5°. This is because the interactions between chitosan and metal ions such as Cr, Mn and Fe could affect the crystallinity via new covalent bonds between the biopolymer and the metal ions^36^. Although several diffraction peaks could be observed in the XRD patterns, the largely widen peaks and weak peak signals demonstrate the amorphous property of these nanofibers.

### Effect of solution pH

**Figure 2:**
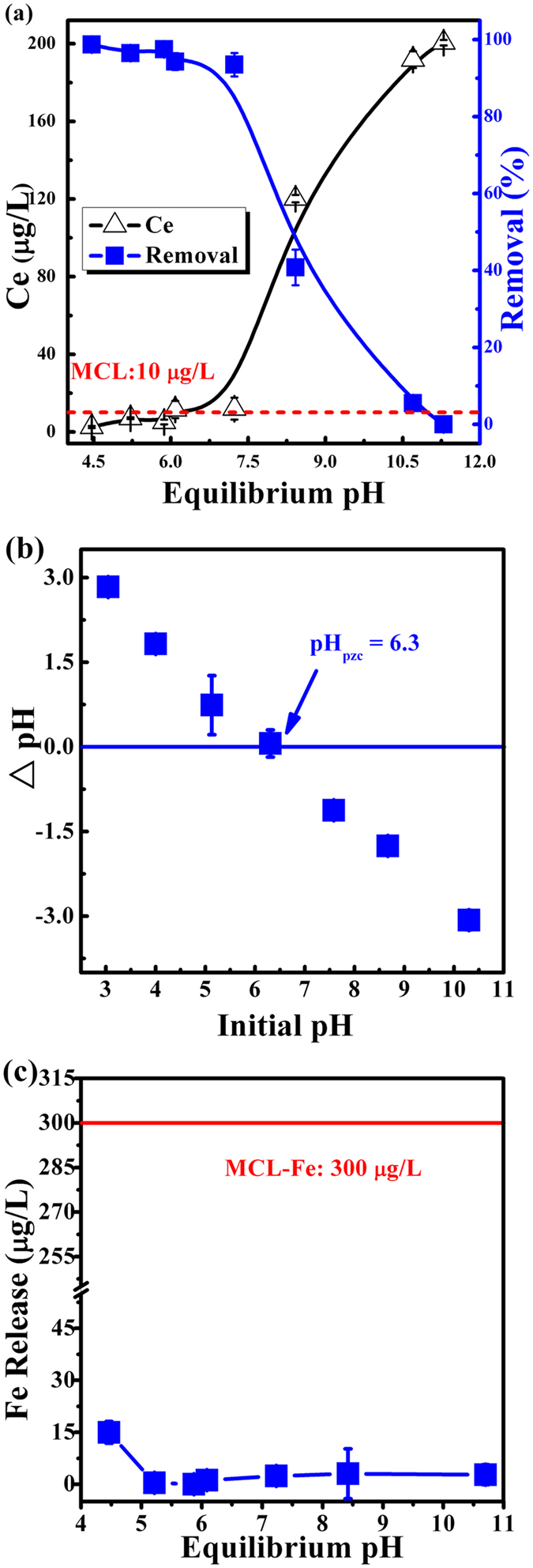
(**a**) Effect of pH on As(V) adsorption by ICS-ENF, (**b**) pH_pzc_ plot of ICS-ENF, and (**c**) iron release during As(V) adsorption (C_0_ = 200 μg/L, adsorbent dose = 0.2 g/L, temperature = 25 °C, contact time = 48 h).

Solution pH could affect the surface charge of adsorbent/adsobate, thus it could play a crucial role in determining the behavior of adsorption system. Figure 2a demonstrates the influence of equilibrium solution pH (from 4.5 to 11.5) on the arsenic adsorption by ICS-ENF. Under the acid and weak basic conditions, ICS-ENF shows effective adsorption of As (more than 90% removal), and adsorption performance decreased slightly from 4.5 to 7.3. This indicates that ICS-ENF (with iron) represented better adsorption capability than that of CS-ENF (without iron), which showed little affinity towards As(V) with the circumstance of pH around 7.0^31^. In addition, ICS-ENF has high adsorption capability at pH = 7, which is very desirable because most natural water bodies are at near neutral condition. However, with the further increasing pH value from a turning-point at 7.3, the adsorption of As(V) dramatically declines and even no adsorption process was observed upon pH up to 11.3. Similar phenomenon has been reported for the adsorption of As(V) by Zr(IV) loaded orange waste gel^1^ and the adsorption of As^37^ by Fe-Mn binary oxide^38^. Such behavior could be explained in terms of pH_pzc_ of the adsorbent in the solution (pH_pzc_ = 6.3, Fig. 2b), as As(V) adsorption would be facilitated by electrostatic interaction between negatively charged As(V) species (H_2_AsO_4_^−^ and HAsO_4_^2−^ are predominant in the experimental pH range) and positively charged absorbent surface. In this case, the higher removal efficiency was due to the abundant protonation of the adsorption sites on ICS-ENF, which interacted with negatively charged arsenate species at pH < pH_pzc_. With the increasing pH, the net surface charge on the adsorbent became less positive and even negative, and repulsive forces between anionic adsorbate and adsorbent which consequently resulted in a decrease of the As(V) adsorption capacity.

Additionally, we also conducted Fe release experiments under various initial pH conditions (Fig. 2c). It is worthwhile to note that no leakage of iron from the implanted nanofiber was observed from pH 5.2 to 10.7, and even at initial pH 3.2, only 17 μg/L of Fe was detected, which is far less than the MCL of Fe in drinking water (300 μg/L). Thus, the results of pH screening and Fe release experiments suggest that this ICS-ENF adsorbent could be suitable for arsenic contaminated ground water treatment.

### Adsorption kinetics and effect of ionic strength

**Figure 3:**
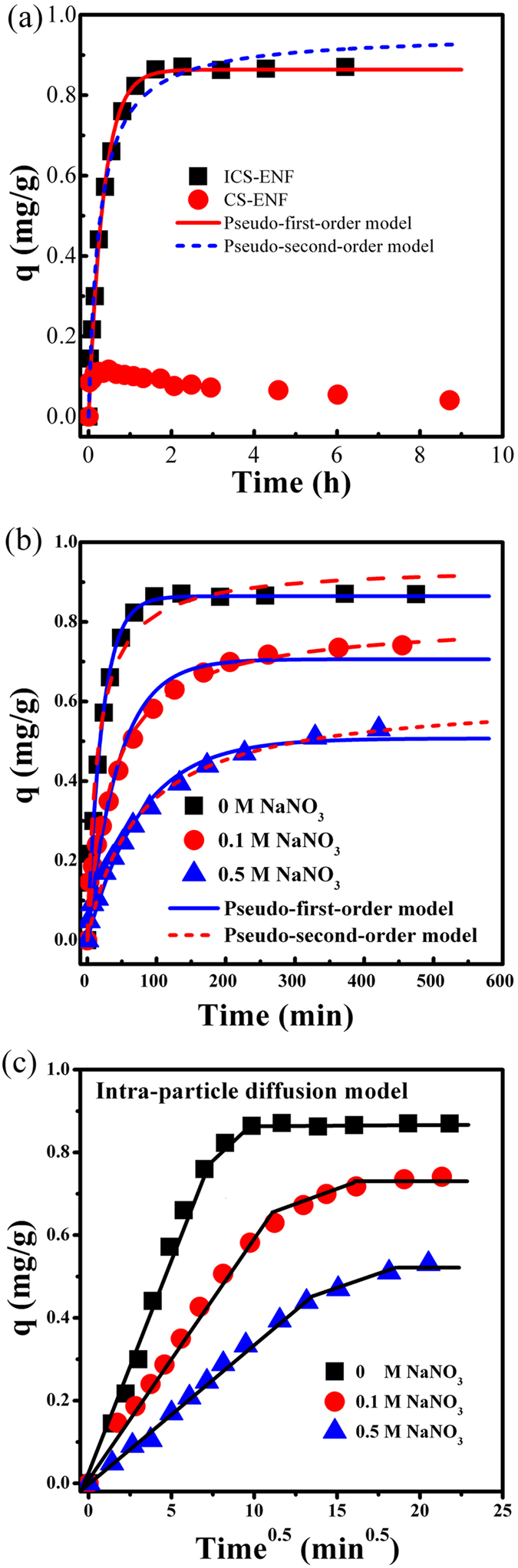
(**a**) As(V) adsorption kinetics on ICS-ENF and CS-ENF, (**b**) Ionic strength effect on As(V) adsorption on ICS-ENF (Adsorbent dose = 0.2 g/L, temperature = 25 °C, final pH = 7.1–7.2), and (**c**) Intra-particle diffusion model for As(V) adsorption on ICS-ENF.

It is very important to be able to predict the adsorption kinetics rate, at which arsenic is removed from contaminated water in order to design appropriate sorption units. Figure 3a shows As(V) adsorption kinetics onto both of ICS-ENF and CS-ENF. It is apparent that the whole adsorption processes could be divided into two stages, a rapid stage at the very beginning following by a gradually slower stage until the adsorption equilibrium was achieved. Typically, the adsorption was nearly reached equilibrium after 100 min and more than 87% of the total adsorption occurred within the first 50 min for ICS-ENF, which was much higher than that of CS-ENF (only 12%, Fig. 3a), and the corresponding maximum adsorption capacities were 0.87 and 0.11 mg/g for ICS-ENF and CS-ENF, respectively. This confirms again that the ICS-ENF with implanted iron significantly improved the As(V) adsorption performance.

The arsenic adsorption mechanism on IC-ENF was first investigated by evaluating the effect of ionic strengths on the adsorption behavior. Figure 3b exhibited that the arsenic adsorption decreased with the increase of solution ionic strength. It implies an outer-sphere adsorption mechanism for As(V) on ICS-ENF, because anions such as NO_3_^−^ as one of potential adsorption competitors, which has been believed to be adsorbed via outer-sphere complexation, are strongly sensitive to ionic strength^39^.

In order to have a better knowledge of the mechanism of arsenate adsorption, the adsorption kinetics data was fitted using different models. Generally, the pseudo-first-order and pseudo-second-order rate equations were frequently employed to analyze the kinetics adsorption data. A linear form of pseudo-first-order kinetics and the pseudo-second-order kinetics model based on the adsorption equilibrium capacity can be mathematically expressed as^35^


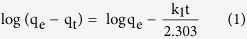



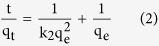


where, q_e_ and q_t_ (mg/g) are the adsorption capacity at equilibrium and at any time t, respectively; and k_1_ (min^−1^) and k_2_ (g mg^−1^ min^−1^) are the rate constants for pseudo-first-order and pseudo-second-order adsorption kinetics model, respectively. Figure 3a,b illustrate the fitting curves of these two models for As(V) adsorption onto ICS-ENF under different ion strength conditions, and the corresponding parameters for pseudo-first-order and pseudo-second-order kinetics models are summarized in Table 1. Based on Table 1, the slightly higher correlation coefficients were observed for pseudo-secondary-order model than that of the pseudo-first-order equations. This indicates chemisorption occurred during As(V) adsorption on the ICS-ENF involving the specific interactions with surface functions group^40^, which will be elucidated more in the following later sections.

Additionally, to further verify the contribution of intra-particle diffusion on the adsorption dynamics process, Weber and Morris model^41^ was also used to analyze the kinetics data of As(V) sorption onto the ICS-ENF (Fig. 3c),


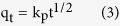


where k_p_ (min^−1^) is the intra-particle diffusion rate constant. If intra-particle diffusion is the rate controlling step, the plot of q_t_ against *t*^1/2^ should give a straight line passing through the origin. However, multi-linearities were clearly observed over the whole time range (Fig. 3c), showing that the three steps could govern adsorption processes. The first linear section exhibited one steep slope refereeing to the rate controlling as the pore diffusion, and the second linear portion was a relatively moderate sorption stage, which indicates the intra-particle diffusion controlling in the pore structure. Finally, the last linear section for slow sorption stage was relating to the stereo-hindrance effect derived from the adsorbed species^42^.

### Adsorption isotherms

**Figure 4:**
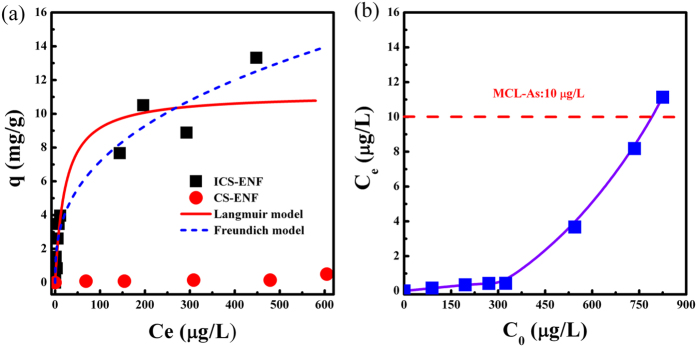
(**a**) As(V) adsorption isotherms on ICS-ENF and CS-ENF, (**b**) Equilibrium As(V) concentration vs. initial As(V) concentrations of ICS-ENF (Final pH = 7.1–7.3, adsorbent dose = 0.2 g/L, temperature = 25 °C, contact time = 48 h).

The arsenic adsorption isotherms for both ICS-ENF and CS-ENF at different initial arsenic concentrations are exhibited in Fig. 4a. ICS-ENF exhibited a maximum capacity of 13.3 mg/g at initial As (V) concentration of 3.1 mg/L (C_e_ 448 μg/L), which was much higher than that (0.5 mg/g) of CS-ENF. This further demonstrates that the doped Fe remarkably improved the As(V) adsorption capacity even under neutral pH conditions. Additionally, Fig. 4b represents the equilibrium As(V) concentration vs. initial As(V) concentration for ICS-ENF adsorption experiments and it indicates that the residual As(V) concentration at equilibrium could be easily decreased to below 10 μg/L (maximum arsenic contaminant level) at initial As(V) concentrations ranging from 100 μg/L to 750 μg/L.

Moreover, two well known adsorption isotherm models namely Langmuir and Freundlich are used to analyze the equilibrium data^8^. Langmuir isotherm assumes monolayer adsorption on a homogeneous surface and the equation is


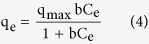


where q_max_ (mg/g) is the maximum amount of arsenate per unit weight of adsorbent, and b (L/mg) is a Langmuir constant which is related to the affinity of the binding sites. q_e_ is the amount of As(V) adsorption corresponding to monolayer coverage. The Freundlich model, which assumes a heterogeneous surface and multilayer adsorption with an energetic non-uniform distribution, is expressed as


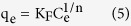


where K_F_ and n are the Freundlich constants.

The adsorption constants obtained from the isotherms for Langmuir and Freundlich models were given in Table S1. As shown in Table S1, both of Langmuir and Freundlich represent well fitting, and the slightly higher regression coefficient suggests that the Freundlich model is more suitable for describing the adsorption behavior of As(V) by ICS-ENF, which indicates that it might be in favor of heterogeneous surface and multilayer adsorption mechanism. Whereas, it is worthwhile to note that the maximum adsorption capacities (q_max_) as estimated by the Langmuir model for ICS-ENF could be up to 11.2 mg/g. Table 2 summarizes the reported adsorption capacities of various adsorbent on arsenic adsorption and ICS-ENF showed a much higher adsorption capacity compared with many other chitosan related adsorbents. In this case, the high arsenic adsorption capacity at low equilibrium arsenic concentrations could benefit the applications of ICS-ENF in real water environment, especially where most contaminated water sources have relatively low arsenic concentrations. Therefore, it can be concluded that ICS-ENF is a promising absorbent for the treatment of low arsenate-containing water including drinking water.

### Effect of coexisting anions

**Figure 5:**
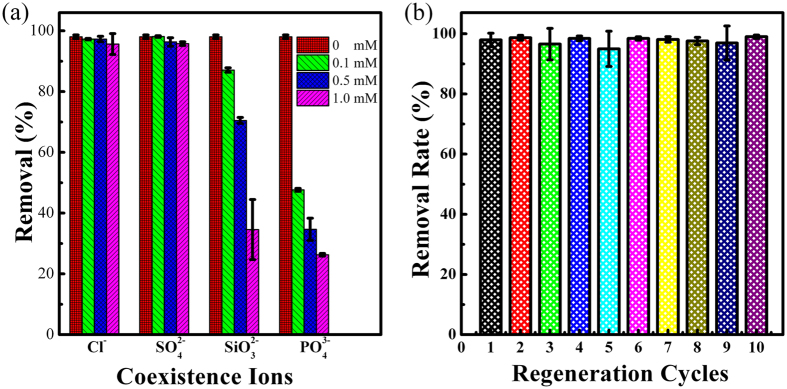
(**a**) Effect of coexistence ions on As(V) removal by ICS-ENF (C_0_ = 200 μg/L (0.003 mM), adsorbent dose = 0.2 g/L, temperature = 25 °C, pH = 7.2, contact time = 48 h. (**b**) Regeneration of ICS-ENF over ten adsorption-desorption cycles with 0.003 M NaOH (C_0_ = 200 μg/L, adsorbent dose = 0.2 g/L, temperature = 25 °C).

Anions ubiquitously present in natural and polluted waters, and can affect the arsenic removal over adsorbent due to the competitive binding to the same adsorption sites between arsenate and anions, thus reducing the arsenate removal. In this study, the representative anions (Cl^−^, SO_4_^2−^, SiO_3_^2−^ and PO_4_^3−^) with concentrations of 0.1, 0.5 or 1.0 mM for each ion were selected as competition anions to study the influence on the adsorption capacity of As(V) with its initial concentration of 0.003 mM (much lower than that of the co-ions). The experimental results (Fig. 5a) indicate that Cl^−^ and SO_4_^2−^ even up to 1.0 mM concentration didn’t have a negative effect on the adsorption of As(V), whereas SiO_3_^2−^ and PO_4_^3−^ could significantly interfere the sorption on ICS-ENF nanofiber. With the concentration of SiO_3_^2−^ and PO_4_^3−^ to 1.0 mM, the removal efficiency decreased up to 34.6% and 26.3%, respectively. The similar adverse effects have been also observed by previous studies^43^,^44^. While it has been reported that the decrease of As(V) sorption for the presence of phosphate might be due to the strong competition as the similar chemical structure with the arsenate^43^, the influence from silicate could be because of itself polymerization which can inhibit arsenate adsorption via steric effects or by decreasing the surface potential of absorbent (Tuutijärvi *et al*.^44^). In this case, the further development of a remediation method for ICS-ENF nanofiber is necessary to overcome the phosphate and silicate negative influence on arsenic adsorption.

### Regeneration and column tests

Regeneration and reusability could be considered as the most important indicators for a potential adsorbent. Generally, it is rather difficult to desorb the arsenate from an aqueous medium, and high concentration sodium hydroxide solution (i.e. 0.1 M) was desirable for stripping arsenic from the adsorbent^8^. However, in the present study, we employed only 0.003 M sodium hydroxide as the desorption solution, and meanwhile the reusability of ICS-ENF was investigated via ten sorption–desorption cycles simultaneously with Fe release monitoring throughout the recycles. As Fig. 5b shown, ICS-ENF still represented the powerful reusability performance with above 98% of As(V) removal efficiency even after ten successive cycles. No obvious change was found in the color, size, weight and shape of the ICS-ENF after 10 cycles of batch adsorption. Furthermore, it is worth to note that no leachable iron was detected during the whole cycling processes. However, a small decrease in the tensile modulus was observed after 10 cycles of batch adsorption, which might be attributed to the partially degradation of chitosan for long time immersion in water. More study should be carried out to further improve the mechanical strength and stability of the ICS-ENF in the future.

Additionally, continuous flow column studies were further employed to examine the ability of ICS-ENF to remove the arsenic from water, which was obtained by pressuring the As(V) solution in an upward flow at a flow rate of 3.6 mL/min and by determining the concentration of arsenic ions at different time intervals in the effluent. The breakthrough curve for As(V) (Fig. S3a) indicates that no arsenic was found in the effluent up to about 300 bed volumes (BV) and even with a fast empty bed contact time (EBCT) of 1.4 min, the ICS-ENF demonstrated a high breakthrough BV of 805 for the treatment of 200 μg/L As(V) under the requirement of the arsenic MCL for drinking water at 10 μg/L. Moreover, by using 0.003 M NaOH stripping, our column regeneration test (Fig. S3b) shows maximum desorption could take place within 4 BV, and about 70 fold enrichment was attained to accomplish the column recycling. Therefore, the results indicate that 0.003 M sodium hydroxide solution could be effective for stripping arsenate from ICS-ENF; and additionally ICS-ENF owns an excellent durability, which can be extensively utilized in water treatment.

### Spectroscopic analysis

The arsenic adsorption mechanism was first investigated by spectroscopic technique of FTIR spectroscopy. Figure S4 demonstrates the FTIR spectra of ICS-ENF before and after batch arsenic adsorption, respectively, where the characteristic absorption bands around at 3364 cm^−1^ (N–H and –OH stretching), 2876 cm^−1^ (C–H stretching), 1642 and 1595 cm^−1^ (N–H bending), 1383 cm^−1^ (–NH deformation), and 1083 cm^−1^ (C–O stretch) conform well to pure chitosan, one of the main components of ICS-ENF^8^,^45^,^46^,^47^. Compared with ICS-ENF alone, the FTIR spectra after arsenic adsorption indicated that the peaks at 3364, 1642, 1383 and 1083 cm^−1^ have weakened, and a new weak band, which is corresponding to As–O stretching vibration, appeared at 844 cm^−1^ 48. This may result from the replacement of Fe bound −OH groups with −OAsO(OH)_2_ moieties^14^. All these observations indicate that confirmed the adsorption of As(V) on the sorbent, and –NH, –OH and C–O could make the dominant contributions for the adsorption of As(V) on ICS-ENF.

In order to further investigate the mechanism of As(V) sorption onto ICS-ENF, binding energy shifts of carbon, nitrogen, oxygen, iron and arsenic were examined using XPS. XPS wide scan spectra of the virgin and arsenate loaded ICS-ENF are illustrated in Fig. S5. Three major peaks at binding energies of 286, 399 and 533 eV, designated for the C 1s, N 1s, and O 1s, respectively, were observed for the adsorbent of ICS-ENF. However, after arsenate adsorption, two new weak peaks at binding energy of about 44.8 eV for As 3d, and 1327.1 eV for As 2p3 appear (Fig. S6), which confirmed the As(V) adsorption onto the ICS-ENF.

**Figure 6:**
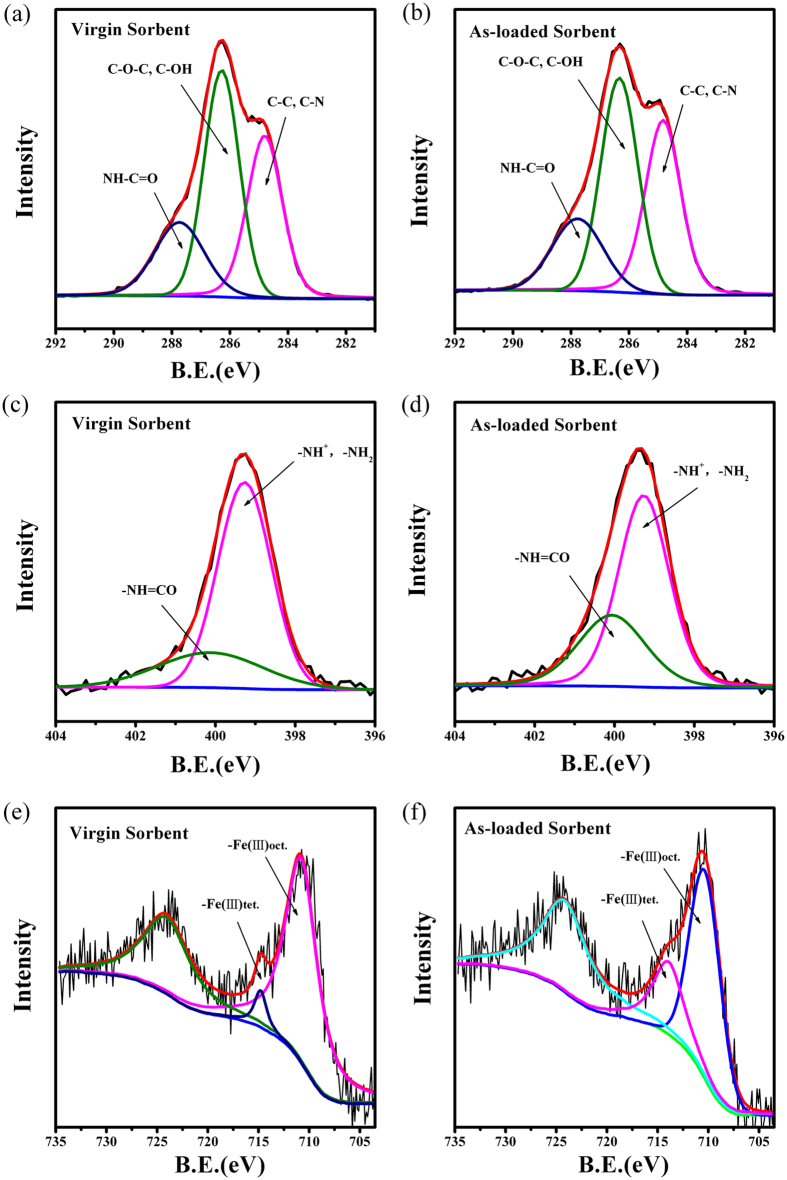
High resolution scan of C 1s XPS spectra of (**a**) fresh ICS-ENF and (**b**) As(V) loaded ICS-ENF, N 1s XPS spectra of (**c**) fresh ICS-ENF and (**d**) As(V) loaded ICS-ENF; Fe 2p XPS spectra of (**e**) fresh ICS-ENF and (**f**) As(V) loaded ICS-ENF (The condition for sample preparation of As(V) loaded ICS-ENF is as below: C_0_ = 10 mg/L, adsorbent dose = 0.2 g/L, temperature = 25 °C, contact time = 48 h).

To have a better knowledge of the structural changes which could be involved in the adsorbent of ICS-ENF during the As(V) adsorption process, we conducted high resolution scan of C 1s, N 1s and Fe 2p XPS spectra (Fig. 6), and the binding energy as well as the relative contents of C 1s, N 1s and Fe 2p were analyzed (Table S2). Based on the Fig. 6 and Table S2, it can be seen that after the As(V) adsorption, the intensity of C–N, NH–C = O and Fe(III)tet increased, while that of C–OH and Fe(III)oct decreased. Therefore, the XPS analysis again suggests that –NH, –OH, C–O and Fe implantation should play important roles in the uptake of As(V), which is in accordance with the above mentioned results during kinetic and FTIR studies^37^. When Fe^3+^ was first dissolved in the chitosan solution, Fe-chitosan complex would formed^49^. And it was concluded that Fe^3+^ was usually coordinated with the functional groups of chitosan, i.e. –NH and –OH^50^. After electrospinning, the nanofibers were dried in air and crossliked by ammonia vapor, and the Fe^3+^ would be converted into iron oxide. When metal oxides were used as As(V) adsorbent, the metal–OH on the surface of the sorbent were formed due to protonation, followed by bonding of anionic As(V)^14^,^51^,^52^. On the basis of all the above analyses, the mechanism of adsorptive removal of As(V) by ICS-ENF is proposed as shown in Fig. S7.

## Conclusions

In summary, an environment-friendly adsorbent, iron doped chitosan nanofiber (ICS-ENF), was successfully synthesized by electrospinning for trace As(V) removal from water. The imagines analysis via XRD, FESEM and TEM coupled with EDX showed the electrospun nanofibers were amorphous, highly porous and fairly uniform. Under acid and weak basic conditions, ICS-ENF shows highly effective As(V) adsorption (more than 90%). Compared with CS-ENF, ICS-ENF demonstrated exceptional adsorption performance in terms of adsorption capacity at neutral pH. The kinetics study exhibited the adsorption was nearly reached equilibrium after 100 min and more than 87% of the total adsorption occurred within 50 min, which could be well fitted by the pseudo-second-order kinetics model. While the arsenic adsorption decreased with increasing solution ionic strength, the coexisting anions of Cl^−^ and SO_4_^2−^ even up to 1.0 mM didn’t cause negative effect on the As(V) adsorption, whereas SiO_3_^2−^ and PO_4_^3−^ could significantly interfere with the sorption on ICS-ENF. The ICS-ENF has a high maximum adsorption capacity of up to 11.2 mg/g at neural pH, and Freundlich model is more suitable for describing the adsorption isotherm Additionally, the spectroscopic analysis implied that –NH, –OH and C–O could make the dominant contributions for the As(V) adsorption on ICS-ENF. Finally, the regeneration and column tests represent that ICS-ENF could be easily regenerated by 0.003 M NaOH solution, and the powerful reusability performance with above 98% of As(V) removal efficiency even after ten successive adsorption-desorption cycles indicated that ICS-ENF could be extensively utilized in arsenic containing water treatment.

## Experimental

### Materials

All chemicals were used as received. Ammonium hydroxide (NH_3_·H_2_O), acetic acid (CH_3_COOH), sodium arsenate (Na_3_AsO_4_·12H_2_O), and ferric chloride (FeCl_3_·6H_2_O) of analytical grade were obtained from Sinopharm Chemical Reagent Co. Ltd. (Shanghai, China). Chitosan with average Mw of 150,000 was supplied by Aoxing Biotechnology Co. Ltd. (Zhejiang, China), and PEO particle with average Mw of 1000,000, was purchased from Changchun Dadi Co. Ltd. (Changchun, China).

### Preparation of adsorbents

The chitosan (4% w/v) and PEO (4% w/v) solutions were separately prepared by dissolving in acetic acid (50% v/v). The chitosan/PEO blend solution for CS-ENF fabrication was obtained by mixing the two master solutions at 14:1 ratios, which was further adjusted for ICS-ENF synthesis with the addition of FeCl_3_ at the ratio of 3 wt% and kept stirring for 5 h prior to the electrospinning. The dosage of FeCl_3_ were determined through a preliminary study. In the preliminary experiments, the amount of FeCl_3_ ranged from 1 wt% to 5 wt%. The result showed that the ICS-ENF with 1 wt% FeCl_3_ doping exhibited a low As(V) adsorption capacity, while 5 wt% FeCl_3_ doping led to poor spinnability of the iron doped chitosan solution. Therefore, 3 wt% FeCl_3_ was chosen as the optimized dosage to fabricate the ICS-ENF.

Nanofiber was fabricated using a simple electrospinning process described previously^31^. Briefly, the as-prepared polymer solution was transferred into a 20 mL syringe coupled with a needle tip (21G), and electrospun under the voltage, tip-collector distance and solution feeding rate of 16 kV, 15.5 cm and 0.9 mL/h, respectively. The fabricated nanofibers were expelled in an air dry oven at 50 °C for 6 h and then crosslinked by ammonia vapor for another 0.5 h. Finally, the CS-ENF and ICS-ENF were obtained after rinsed with deionized water and dried at 30 °C, which were used as adsorbents for arsenate adsorption.

### Characterization of the nanofibers

The surface morphology of the nanofibers was obtained by a Field Emission Scanning Electron Microscopy (FESEM, Hitachi S4800, Japan). The transmission electron micrographs (TEM) and the selected area electron diffraction micrographs were taken with a Tecnai F20 transmission electron microscope (Phillips, USA) operating at 200 kV. Mechanical strength of the electrospun nanofibers was determined using a tensile machine (AGS-X, Shimadzu, Japan). X-ray diffraction (XRD) patterns were recorded with an XRD System model X’ Pert PRO (PANalytical, Netherlands) using a Ni filter, Cu Ka radiation (λ = 1.54060A) and angular variation of 570° (2θ).

The functional groups on the surface of ICS-ENF were characterized by Fourier transform infrared spectroscopy (FTIR, Thermo NICOLET iS10, USA) measurements using transmission mode, with wavelengths in the range from 200 to 4000 cm^−1^. XPS (PHI Quantum 2000, USA) with X-ray source of Mg Ka was used to determine the chemical composition of the nanofiber adsorbent before and after arsenic uptake. For wide scan XPS spectra, an energy range from 0 to 1200 eV was used with pass energy of 80 eV and step size of 1.6 eV. The high resolution XPS scans were conducted according to the peak being examined with pass energy of 40 eV and step size of 0.125 eV. The XPS results were collected in binding energy forms and fitted using a nonlinear least squares curve fitting program. Meanwhile, the carbon 1s electron binding energy corresponding to graphitic carbon at 284.8 eV was used as reference for calibration purposes.

The point of zero charge (pH_pzc_) of the ICS-ENF was determined via batch equilibration technique. NaNO_3_ solution with the concentration of 0.1 M was used as the electrolyte maintaining the constant ionic strength in all experiments which was adjusted to various pH values from 3.0 to 10.0 by diluted HCl or NaOH. After the 48-hour agitation, the final pH values (pH_final_) were measured and the pH_pzc_ of the nanofiber adsorbent was accordingly yielded based on the plot of ΔpH (pH_final_–pH_initial_) against pH_initial_.

### Batch adsorption experiments

All the batch adsorption experiments were performed with a fixed absorbent dosage of 0.2 g/L at 25 °C. Arsenate stock solution (1000 mg/L) was prepared by dissolving exact amount of Na_3_AsO_4_·12H_2_O into ultrapure water, which is further diluted for the desired concentration. After shaking for a predetermined time, the equilibrium arsenate solution, was filtered through 0.45 μm filter membranes, and then determined by ICP-MS (Agilent 7500cx, USA). In this case, the amount of As(V) adsorbed^53^ per unit mass of adsorbent (g), q_e_ (mg/g), was obtained by the following equation:


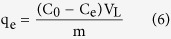


where C_0_ and C_e_ denote the initial and equilibrium concentration of As(V) (mg/L), respectively. V_L_ is the volume of the adsorption solution (L), and m is the weight of dry adsorbents (g).

To examine the effect of pH on the adsorption process, 8 mg of adsorbent was mixed with 40 mL As(V) solution at the initial pH values of 3.2–11.4 (adjusted by 0.1 M HCl and 0.1 M NaOH) for 48 hour agitation, and the final equilibrium As(V) concentration and pH values were accordingly analyzed.

Kinetics studies coupled with the effect of ionic strength, which was adjusted via NaNO_3_ solution at the concentration ranging from 0 to 0.5 M, were conducted by adding 40 mg ICS-ENF into 200 mL arsenic solution (200 μg/L). The samples were withdrawn at appropriate time intervals and analyzed by the ICP-MS.

The adsorption isotherm experiments were performed by using initial arsenic concentrations ranging from 0.1 to 3.1 mg/L. Additionally, four representative anions (Cl^−^, SO_4_^2−^, SiO_3_^2−^ and PO_4_^3−^) with concentration ranging from 0.1 to 1.0 mM were used to investigate the effect of competing anions on arsenate adsorption.

### Regeneration and column adsorption experiments

The adsorption-desorption (regeneration) experiments were undertaken by mixing 10 mg of ICS-ENF with 50 mL As(V) solutions (200 μg/L). After 3 h of agitation, the adsorbent was extracted, washed and transferred to a new bottle. Then, 50 mL of the 0.003 M NaOH solution was added and shaken for another 3 h. The solution was filtered and the arsenic concentration was determined in the filtrate. Additionally, followed by the above mentioned regeneration protocols, ten cycles of adsorption-desorption experiment were carried out to evaluate the reusability of ICS-ENF for arsenic removal.

Continuous flow column tests were conducted to examine the performance of arsenic removal by ICS-ENF for its potential applications. A glass tube with 0.9 cm inner diameter and 10 cm length packed with 0.7 g ICS-ENF was used to conduct the flow column experiment with an up-flow pattern. The flow rate was kept at 3.6 mL/min and the pH of the influent solution was 7.2. Elution test was conducted by 0.003 M NaOH and the concentration of arsenic and iron concentration were analyzed for the effluent solutions from the column.

## Additional Information

**How to cite this article**: Min, L.-L. *et al*. Functionalized chitosan electrospun nanofiber for effective removal of trace arsenate from water. *Sci. Rep*. **6**, 32480; doi: 10.1038/srep32480 (2016).

## Supplementary Material

Supplementary Information

## Figures and Tables

**Table 1:** Parameters of pseudo-first-order, pseudo-second-order & intro-particle diffusion model of adsorption kinetics of arsenic on ICS-ENF.

[NaNO_3_]	Pseudo-first-order			Pseudo-second-order			Particle diffusion model	
	q_e_ (mg/g)	k_1_ (min^−1^)	R^2^	q_e_ (mg/g)	k_2_ (g/(mg min))	R^2^	K_p_ (mg/(g min^0.5^))	R^2^
0 mM	0.860	0.047	0.992	1.026	0.052	0.994	0.111	0.992
0.1 mM	0.706	0.022	0.968	0.806	0.035	0.986	0.063	0.992
0.5 mM	0.507	0.013	0.982	0.616	0.023	0.993	0.034	0.991

**Table 2:** Maximum adsorption capacity (mg/g) of As(V) on chitosan related adsorbents reported in literature.

Adsorbent	Max [As]_0_ (mg/L)	pH	q (mg/g)	Ref.
CS*	0.5	5.0	0.5	54
CS/Clay/Magnetite	336	5.0	6.5	35
TiO_2_/CS beads	10	7.0	4.9	55
Magnetite/Graphene	7	7.0	5.8	34
Zero valent iron/CS fiber	25	6.0	2.3	17
CS-ENF	0.7	7.2	0.5	This work
ICS-ENF	1.8	7.2	11.2	This work

^*^Chitosan.
