# Supplementary material for: Functionalized chitosan electrospun nanofiber for effective removal of trace arsenate from water
Source: Sci Rep. 2016 Aug 30;6:32480. doi: 10.1038/srep32480 (PMC5004125; doi:10.1038/srep32480)
Supplement: Supplementary Information [file srep32480-s1.doc]

**Supporting Information**

**Functionalized chitosan electrospun nanofiber for effective removal of trace arsenate from water**

Ling-Li Min^1,2^, Lu-Bin Zhong^1^, Yu-Ming Zheng^1,*^, Qing Liu^1,2^, Zhi-Hua Yuan^1^ & Li-Ming Yang^3,*^

^1^CAS Key Laboratory of Urban Pollutant Conversion, Institute of Urban Environment, Chinese Academy of Sciences, Xiamen 361021, China

^2^College of Resources and Environment, University of Chinese Academy of Sciences, Beijing 100049, China

^3^Department of Chemical & Biomolecular Engineering, National University of Singapore, 21 Lower Kent Ridge Road, 119077, Singapore

^*^Correspondence and requests for materials should be addressed to Y.M.Z. (email: ymzheng@iue.ac.cn) or L.M.Y. (cheylm@nus.edu.sg).

| Langmuir | | | Freundlich | | |
| --- | --- | --- | --- | --- | --- |
| q_max_ (mg/g) | b (L/mg) | R^2^ | K_F_ (mg/g) | 1/n (mg/L)^n^ | R^2^ |
| 11.19 | 0.045 | 0.916 | 1.301 | 0.372 | 0.953 |

**Table S1. Parameters of Langmuir and Freundlich models for As(V) adsorption isotherm on ICS-ENF.**

**Table S2. Binding energies and relative contents of C, N and Fe in the fresh and As(V)-loaded ICS-ENF.**

| Valence state | Sample | Assignment | Binding energy (ev) | Intensity (counts/s) | Relative Content (%) |
| --- | --- | --- | --- | --- | --- |
| C 1s | ICS-ENF | C-N, C-C | 284.8 | 4187.0 | 7.1 |
|  |  | C-OH, C-O-C | 286.3 | 5613.0 | 88.4 |
|  |  | NH-C=O | 287.7 | 2109.2 | 4.5 |
|  | As loaded ICS-ENF- | C-N, C-C | 284.8 | 4584.6 | 36.8 |
|  |  | C-OH, C-O-C | 286.3 | 5576.1 | 43.1 |
|  |  | NH-C=O | 287.8 | 2205.1 | 20.0 |
| N 1s | ICS-ENF | -NH_2_, -NH^+^ | 399.3 | 2248.1 | 74.9 |
|  |  | NH-C=O | 400.1 | 1188.1 | 25.1 |
|  | As loaded ICS-ENF | -NH_2_, -NH^+^ | 399.4 | 2228.0 | 77.5 |
|  |  | NH-C=O | 400.2 | 1533.0 | 22.5 |
| Fe 2p | ICS-ENF | Fe(Ⅲ) oct. | 710.8 | 2713.1 | 93.7 |
|  |  | Fe(Ⅲ) tet. | 714.8 | 2441.5 | 6.3 |
|  | As loaded ICS-ENF | Fe(Ⅲ) oct. | 710.3 | 2791.3 | 67.0 |
|  |  | Fe(Ⅲ) tet. | 713.9 | 2616.9 | 33.0 |

| 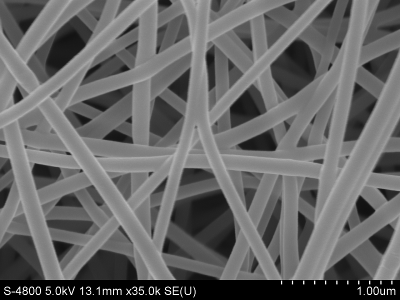 | 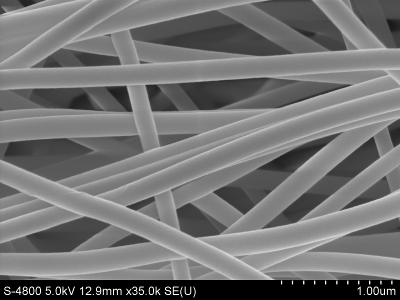 |
| --- | --- |
|  | |

**Figure S1.** FESEM images and fiber diameter distribution of (a) CS-ENF, and (b) ICS-ENF. **(c)** Stress–strain curves of the CS-ENF and ICS-ENF.

**
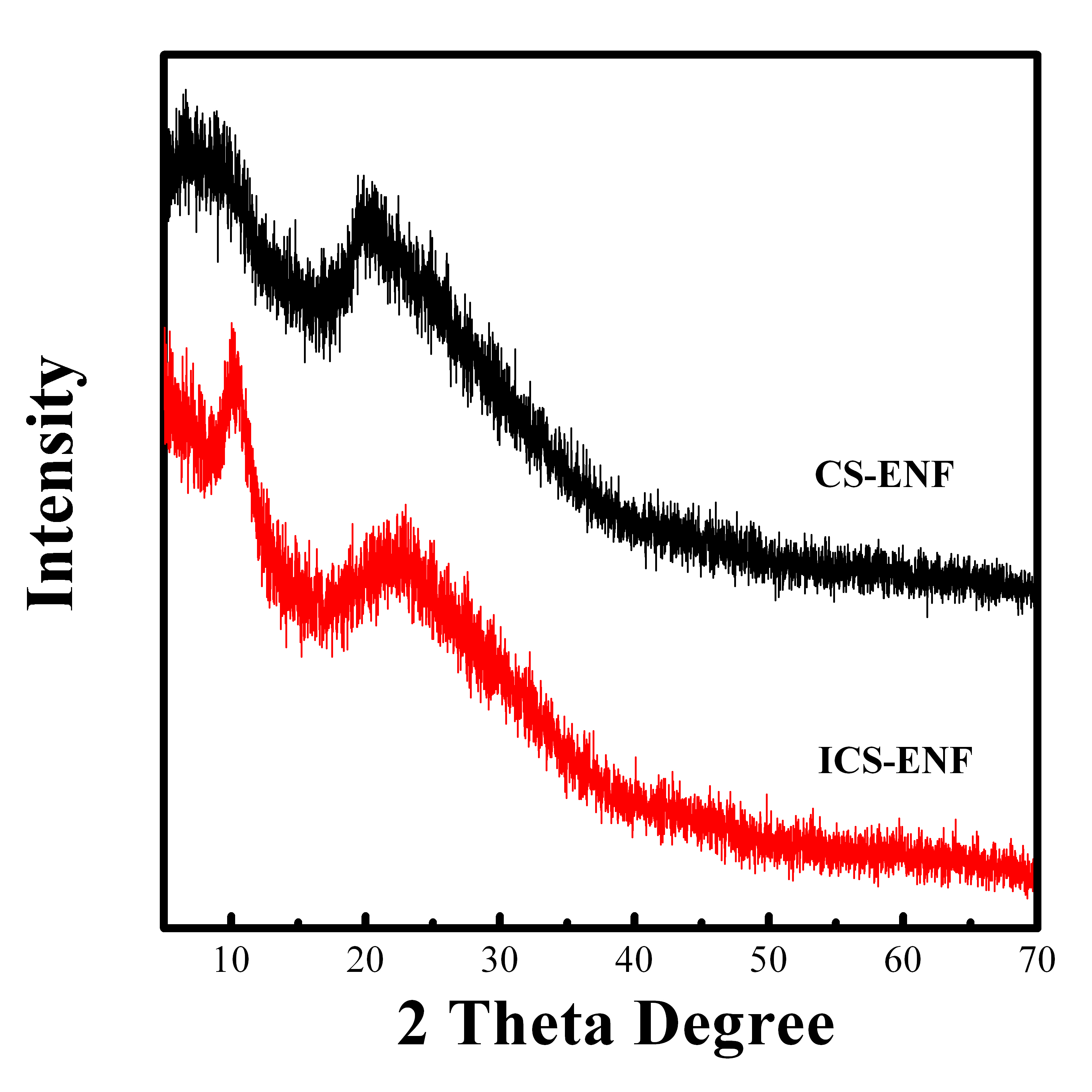
**

**Figure S2.** X-ray diffraction patterns of CS-ENF and ICS-ENF.


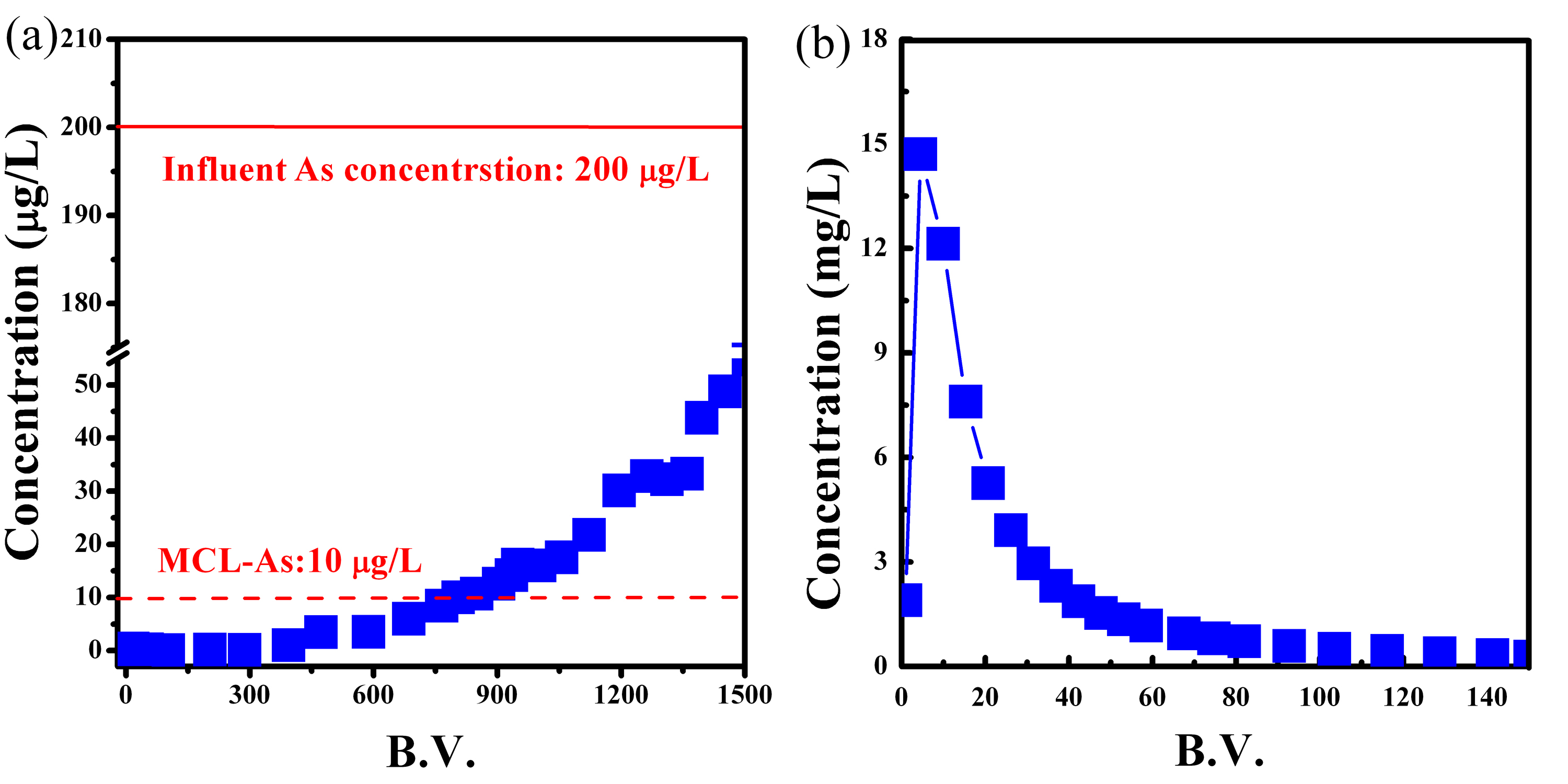


**Figure S3.** (a) Breakthrough curve for adsorption of As(V) on the ICS-ENF (C_0_ = 200 µg/L, flow rate = 3.6 mL/min, pH = 7.2, bed volume = 5 mL), and (b) Regeneration of the ICS-ENF loaded with As(V) using 0.003 M NaOH.


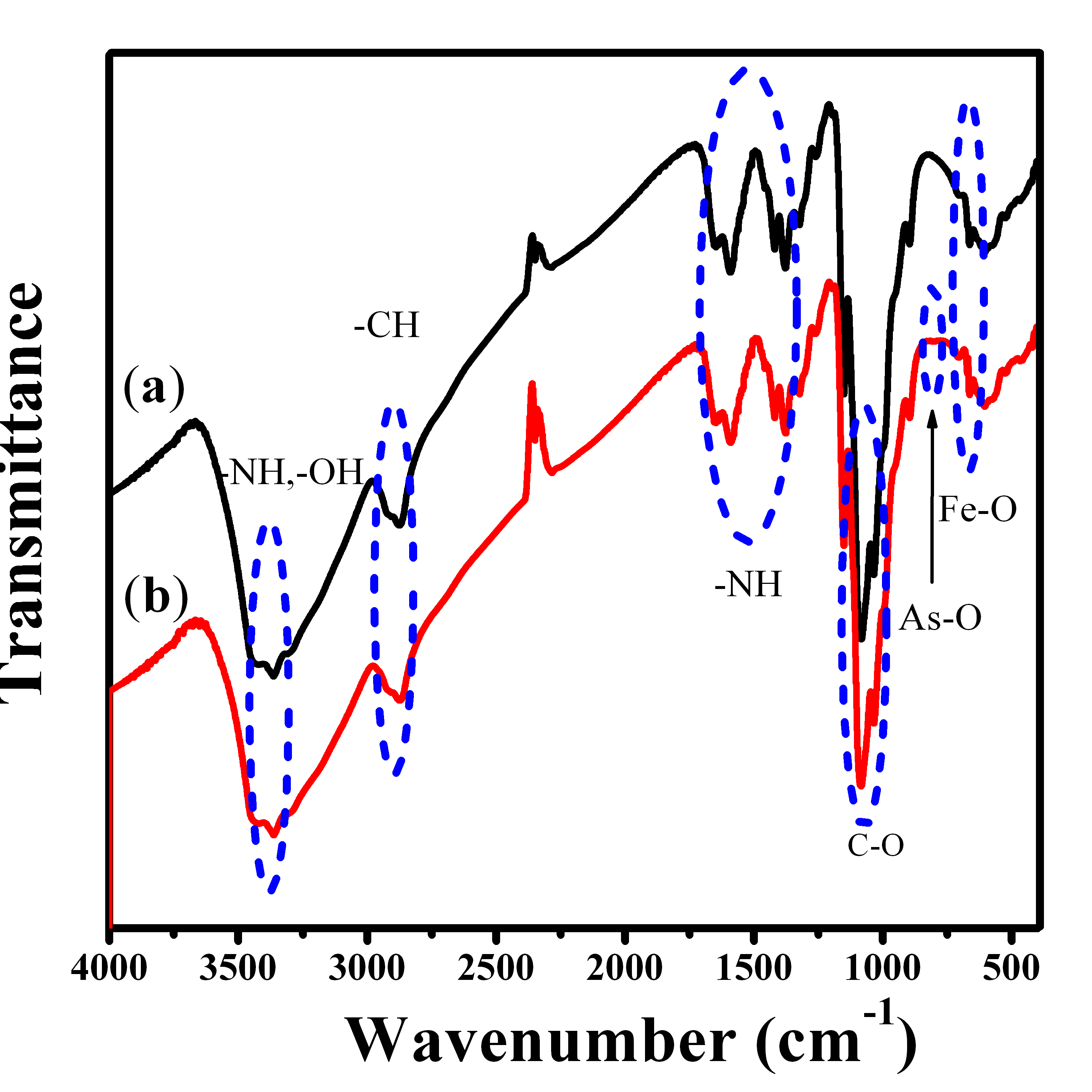


**Figure S4.** FTIR spectra of (a) fresh ICS-ENF, and (b) As(V) loaded ICS-ENF. (The condition for sample preparation of As(V) loaded ICS-ENF is as below: C_0_ = 10 mg/L, adsorbent dose = 0.2 g/L, temperature = 25 °C, contact time = 48 h).


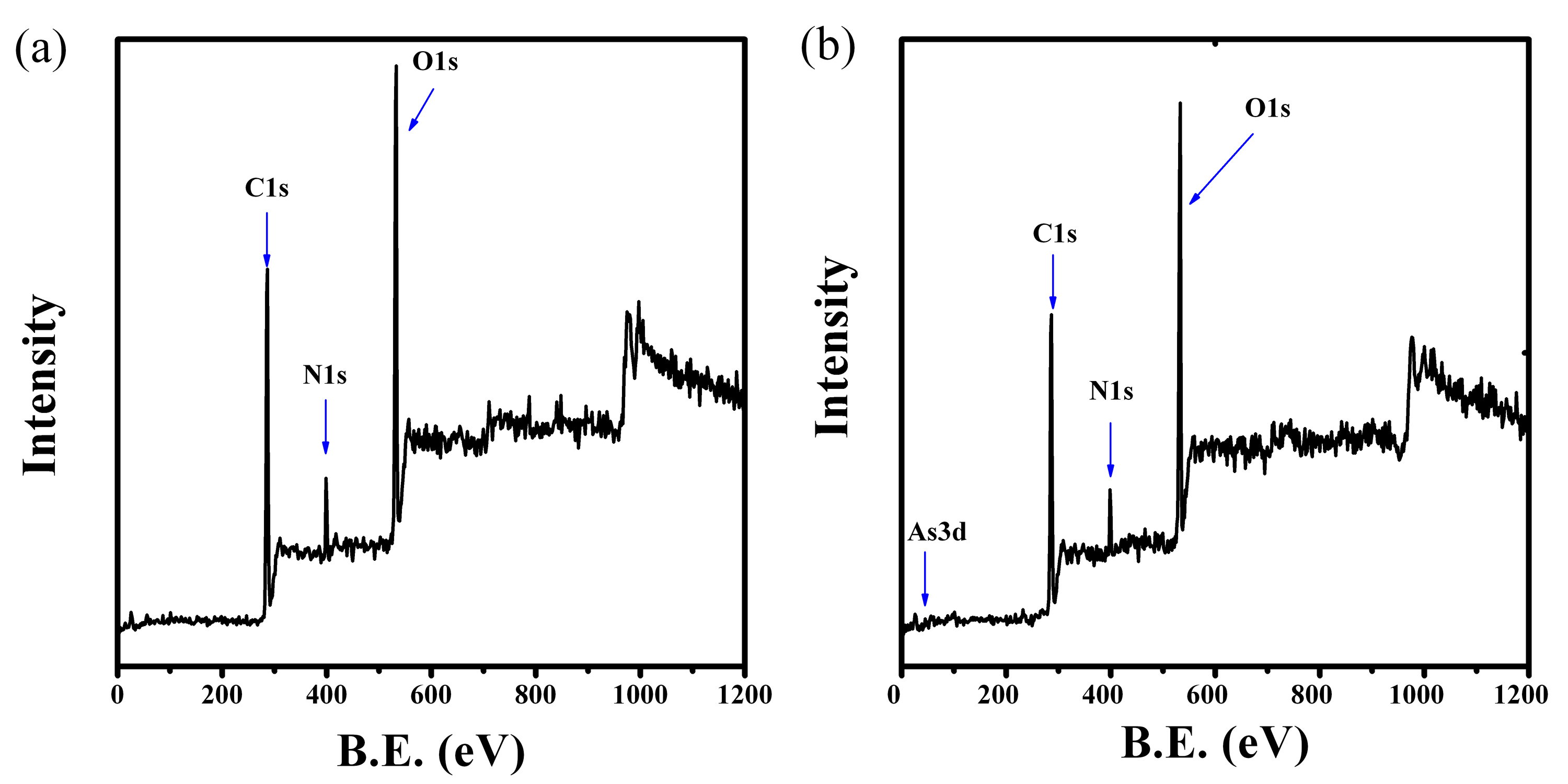


**Figure S5.** XPS wide scan spectra of (a) fresh ICS-ENF, and (b) As(V) loaded ICS-ENF.


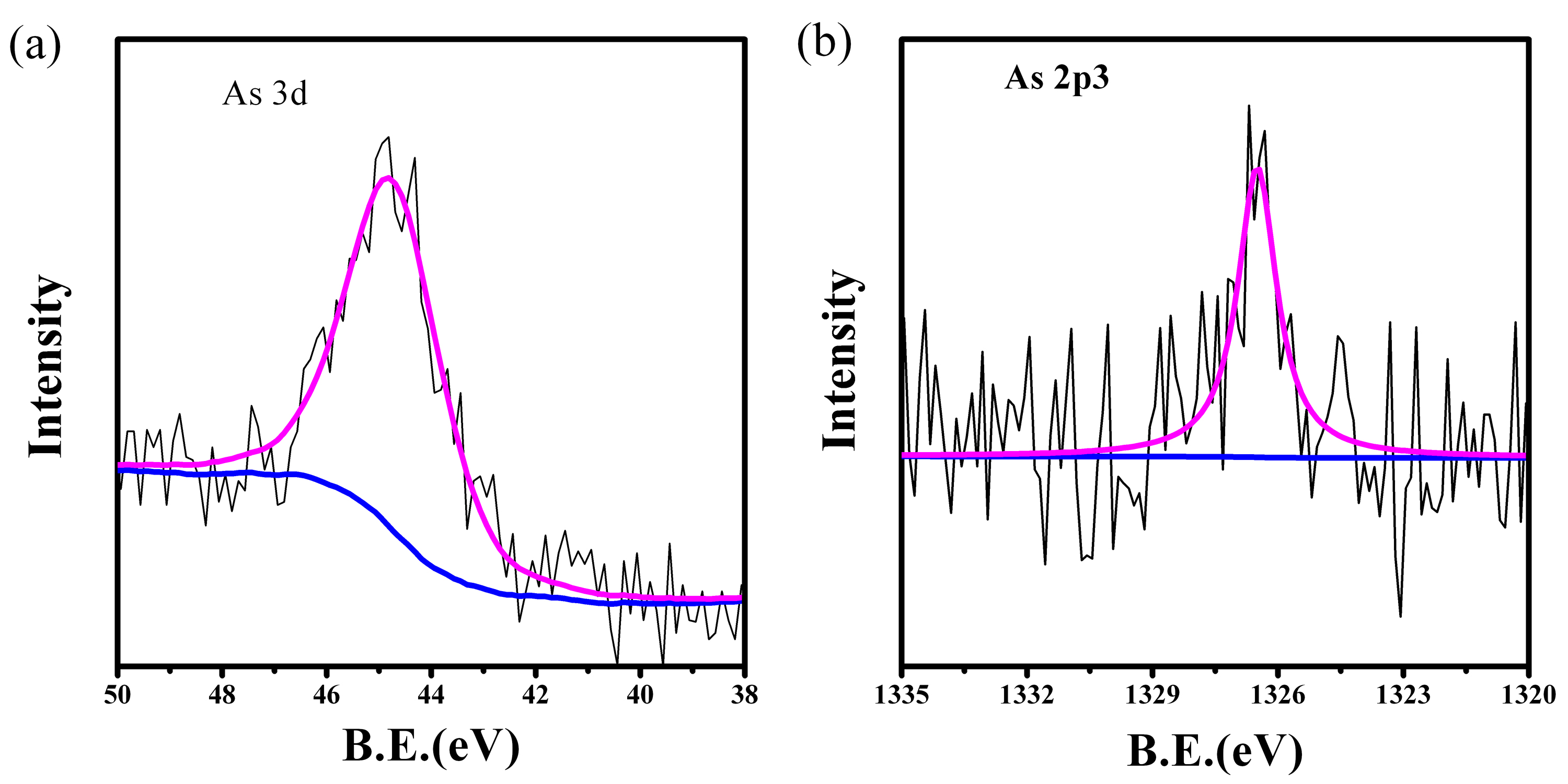


**Figure S6.** High resolution scan of (a) As 3d XPS, and (b) As 2p3 XPS spectra of As(V) loaded ICS-ENF.


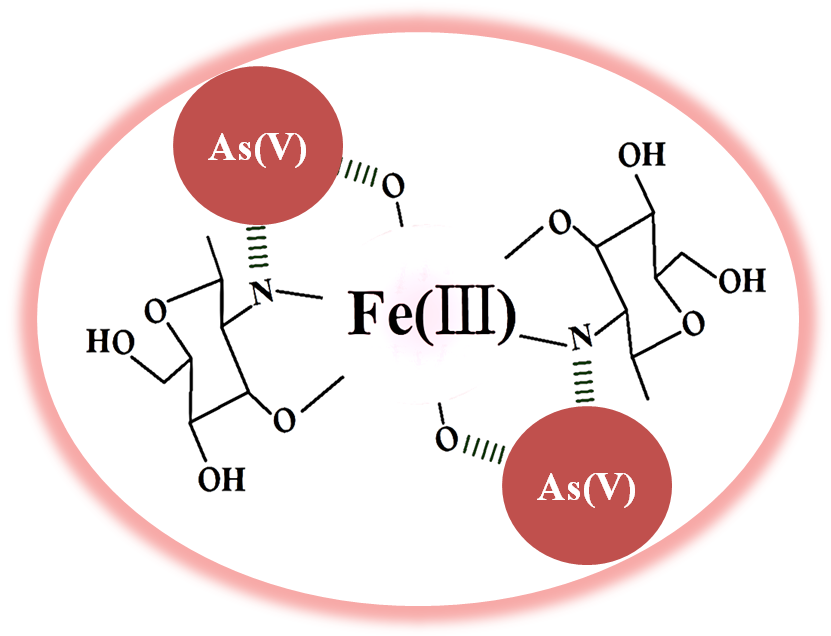


**Figure S7.** Schematic diagram of mechanism for adsorption of As(V) on the ICS-ENF.
